# Patient-Provider Matching, Engagement, and Outcomes of a Digital Mental Health Treatment Platform: Real-World Retrospective Cohort Study

**DOI:** 10.2196/81121

**Published:** 2026-01-23

**Authors:** Valerie L Forman-Hoffman, Edward Hsyeh, Manoj Kanagaraj, Alexander Gille, Matthew Ceneviva, Cynthia Grant

**Affiliations:** 1 Grow Therapy New York, NY United States

**Keywords:** internet, psychotherapy, provider selection, anxiety, depression

## Abstract

**Background:**

Technology-enabled mental health platforms that incorporate user-driven patient-provider matching may offer a novel way to personalize and optimize outcomes. We conducted this study because little is known about the engagement and clinical symptom changes of these newer types of mental health platforms and whether patient-driven selection of their provider’s characteristics is associated with either engagement or clinical outcomes.

**Objective:**

This study aimed to determine the levels of engagement and clinical symptom changes associated with the use of a technology-enabled mental health platform that allows patients to select preferred provider characteristics and to explore whether the selection of a provider characteristic was associated with engagement and clinical outcomes.

**Methods:**

We conducted a real-world, retrospective cohort study using deidentified electronic health data from adult Grow Therapy patients aged 18 years or older with clinically elevated depressive or anxiety symptoms at baseline (PHQ-9 [Patient Health Questionnaire-9] > 9 or GAD-7 [Generalized Anxiety Disorder-7] > 9). Inclusion required 1 provider visit (intent-to-treat cohort) for engagement analyses; clinical outcome analyses required 2 or more provider visits (complete case cohort). Engagement with the platform was measured by the number of provider visits. Clinical outcomes were measured using changes in PHQ-9 and GAD-7 scores and defined as meeting a minimal clinically important difference (MCID). Bivariate associations between selection of provider characteristics and outcomes were measured using chi-square tests, and adjusted associations were modeled using logistic regression (*P*<.05).

**Results:**

Among 159,448 patients with elevated depressive symptoms and 167,356 patients with elevated anxiety symptoms, engagement was high, with 69.4% (95% CI 69.2%-69.7%) and 69.3% (95% CI 69.1%-69.5%) having 3 or more visits, respectively. In the complete case cohort, symptom reductions were significant; 58.9% (95% CI 58.5%-59.2%) met depressive symptom MCID criteria, and 63% (95% CI 62.6%-63.3%) met anxiety symptom MCID criteria after engagement. Although only ≈35% of patients selected a provider specialty and ≈5% selected a provider identity before enrollment, those selecting a provider specialty experienced significantly better outcomes, and those selecting a provider identity engaged significantly more frequently as compared to those who did not select each characteristic. Sensitivity analyses confirmed these findings.

**Conclusions:**

This exploratory, real-world, uncontrolled study provides early evidence that allowing patients to select provider characteristics within a technology-enabled mental health platform may support both engagement and meaningful symptom improvement. The investigation of the relationship between mental telehealth provider selection characteristics and both engagement and clinical outcomes is a novel addition to the peer-reviewed literature. Findings highlight how user-driven, scalable matching features may personalize mental health care in ways that differ from traditional assignment-based models and underscore the need for more rigorous, controlled studies to demonstrate efficacy and test causality and mechanisms.

## Introduction

Despite major advances in mental health care delivery over the past several decades, effectiveness rates have remained modest [1-4]. Few interventions have enabled patients to sustainably engage [5-7], despite the development of web-based tools that have improved access. Nonetheless, the increased demand for flexible and scalable types of psychological support has enabled digital mental health platforms to rapidly expand their reach [8,9].

Some of these platforms offer patients greater choice than is typically available in traditional care settings, such as the option to select providers based on specific personal or professional characteristics. At the same time, however, there is growing recognition that engagement remains one of the most persistent challenges in digital mental health care, with many users disengaging early or never initiating care at all [6,7,10]. Whether the selection of provider characteristics by patients is associated with engagement or clinical outcomes, however, has yet to be reported. Prior research suggests that aspects of provider-patient concordance, such as shared cultural background, language, gender identity, or therapeutic orientation, have the potential to influence therapeutic alliance, treatment satisfaction, and engagement [11,12]. Accordingly, a randomized controlled trial (RCT) found that matching patients to providers based on therapists’ performance strengths can improve mental health outcomes [13]. Previous studies, however, mostly come from traditional psychotherapy contexts that have limited generalizability to digital environments. These shifts highlight the urgent need to better understand how patients make choices within digital ecosystems and whether those choices relate meaningfully to engagement or clinical benefit.

With the growth of digital and hybrid care models, reimbursement for telehealth services, and direct-to-consumer platforms, understanding how patients make provider choices and how these choices relate to the course of treatment becomes increasingly important [14-16]. Technology-enabled platforms that incorporate user-driven matching may offer a novel way to personalize and optimize engagement between users and providers [17]. Some of these platforms, such as Grow Therapy, further empower patients to self-select preferred characteristics of their provider, but little is known about these selections and their associations with engagement and clinical outcomes.

Findings of prior studies that have investigated similar relationships, such as whether patients who visit providers with the same gender or race/ethnicity experience improved outcomes, have had mixed findings [18], with little clarity regarding whether the match characteristics were a random occurrence or patients actually had the choice to select provider characteristics. Fortunately, digital platforms now operate at sizes large enough to evaluate naturally occurring behaviors and outcomes using real-world data, offering an opportunity to study how the autonomy of patients to select their own providers may influence their care trajectories. As such, this study addresses a key gap in the literature by examining a modern digital platform in which patients, rather than systems, have better control over the selection of their provider-matching experience, given that typical access barriers, such as the availability of providers in a particular location, are removed.

Despite the noted benefits of personalization and patient choice central to web-based mental health models [19], empirical evidence on how users navigate provider-selection tools is remarkably sparse. Existing work has largely focused on algorithmic matching systems or high-level engagement metrics, leaving major questions unanswered regarding the real-world behaviors of users who manually browse and select providers [20-22]. Moreover, no web-based mental health care studies to date have examined whether specific provider characteristics, such as clinical expertise, demographics, or therapeutic style chosen by patients, are associated with subsequent engagement patterns or symptom change.

Understanding these patterns is particularly critical given the current emphasis on equity-centered care and culturally responsive provider matching [23,24]. Without foundational descriptive data on how patients actually use these features, platforms lack evidence to guide interface design, optimize provider profiles, or enhance matching systems in ways that support adherence and therapeutic outcomes. This study addresses this gap by offering an empirical examination of provider-selection behaviors and downstream engagement within a large, diverse sample of real-world digital mental health users. Understanding whether selecting specific provider characteristics is meaningfully associated with patient engagement or clinical improvement can clarify how digital platforms might guide patients toward choices that optimize therapeutic fit, strengthen the care experience, and ultimately improve engagement and clinical outcomes.

The specific aims of this study were to (1) describe age, gender, and clinical characteristics of patients enrolled in one such technology-enabled platform created to connect patients to care (Grow Therapy) and (2) determine patients’ levels of engagement (primary) and clinical outcomes (secondary), namely changes in symptoms of depression and anxiety between the first and last visit in this real-world, retrospective cohort study while recognizing the sources of potential biases in interpretation of the findings. A third, exploratory aim was to determine whether there were significant associations between 2 types of provider selection (selection of a provider’s specialty and selection of a provider’s identity characteristic) and meaningful (1) engagement and (2) clinical outcomes that may serve as preliminary signals that can suggest the conduct of additional research studies with more rigorous, hypothesis-testing designs. These additional studies could be used to more definitively determine whether patient selection of provider characteristics, an emerging, scalable feature of digital platforms, can improve engagement or influence clinical outcomes.

## Methods

### Study Design

This study used a real-world, retrospective cohort design leveraging deidentified electronic health record data from Grow Therapy, a technology-enabled mental health platform, reported in accordance with STROBE (Strengthening the Reporting of Observational Studies in Epidemiology) guidance for observational studies [25]. Adults aged 18 years or older with a first provider visit between January 1, 2022, and April 30, 2024, were eligible for inclusion. Patients were included in the intent-to-treat cohorts if they had clinically elevated symptoms at intake (PHQ-9 [Patient Health Questionnaire-9 item]>9 for the depressive symptom cohort; GAD-7 [Generalized Anxiety Disorder]>9 for the anxiety symptom cohort). Clinical outcome analyses used complete case cohorts requiring at least 2 completed PHQ-9 or GAD-7 assessments. Sample size was determined by the available population meeting these criteria, consistent with retrospective observational designs. Data included patient demographics, preferred provider characteristics selected during the matching process (specialty and identity attributes), number of completed visits, and routinely collected symptom assessments. All symptom data originated from standardized, validated electronic instruments integrated into the platform and administered at intake and approximately every third visit. No new data were collected for research purposes. Participants self-selected providers via a predefined interface, and all data used in this study were originally collected as part of routine clinical care.

### Ethical Considerations

This study was reviewed by Western Institutional Review Board Copernicus Group and deemed exempt under 45 Code of Federal Regulations §46.104(d)(4) (Confirmation ID 45689720), which covers secondary research using identifiable private information or biospecimens when data are recorded in a manner that no longer allows subjects to be identified. All individuals agreed to Grow Therapy’s privacy policy and informed consent procedures at intake, which permitted the use of deidentified data for research purposes. As this study involved secondary analyses of deidentified data, additional participant consent was not required. All data were extracted in deidentified form, with direct identifiers removed before analysis. Data were stored in HIPAA (Health Insurance Portability and Accountability Act)-compliant secure databases, and only aggregated results are reported. No compensation was provided to individuals whose clinical data were included in this secondary analysis. No images or materials containing individually identifiable patient information are included in this paper or supplementary materials. All analyses were conducted on deidentified data, which precluded the identification of individual participants.

### Study Design and Sample Identification

Data on all adult patients (18 years or older) with a first Grow Therapy visit between January 1, 2022, and April 30, 2024, were included in this study’s sample, which was further broken down into 2 cohorts. The first cohort consisted of patients who had elevated levels of depressive symptoms, and the second cohort consisted of patients who had elevated levels of anxiety symptoms as described below. Data included preferred provider characteristics endorsed by the patient when seeking to find a new provider affiliated with Grow Therapy and deidentified clinical data collected as part of treatment engagement with a provider, as well as the total number of visits between the patient and provider. Data collected through April 30, 2025, were included in analyses to allow all patients to have at least 12 full months of follow-up.

### Variables of Interest

#### Patient Demographic Characteristics

Patient age at the time of appointment was examined continuously and also categorized into age groups (18-24, 25-34, 35-44, 45-54, 55-64, and 65+ years). Patient self-reported gender was coded into 3 groups as male, female, or other/nonbinary/preferred not to answer.

#### Patient Selection of Provider Characteristics

Types of provider selection characteristics selected by patients included (1) provider specialty (eg, clinical issues such as depression or anxiety, trauma, family conflict, etc), and (2) provider identity (eg, race/ethnicity, religious affiliation, and sexual orientation). Provider identity characteristics refer to self-reported demographic or cultural attributes made visible to patients during the provider selection process. Both of these characteristics were coded as yes or no to indicate whether the patient had chosen a provider specialty or provider identity, respectively, before engaging in a Grow Therapy patient-provider relationship. Patients selected from a standardized list of provider characteristics presented through the Grow Therapy matching interface during intake. These options were not free-text but offered as predefined identity and specialty attributes of each provider.

#### Engagement

The total number of individual, couples, or family outpatient psychotherapy, medication evaluation, or medication management noncancelled visits (ie, number of visits) was quantified and then dichotomized into 2 variables for analyses. First, a variable for having 3 or more visits was used as a reasonable benchmark by which to establish an initial relationship between patient and provider with peak therapeutic alliance that has been shown to lead to better outcomes [26], and, second, a variable quantifying having 12 or more visits benchmarked the length of a typical cognitive behavioral therapy (CBT) intervention.

### Clinical Outcomes

#### Symptoms of Depression

The PHQ-9, a validated and reliable measure of symptoms of depression (Cronbach α=0.86-0.89) [27], was administered electronically to patients at the start of treatment as well as before every third visit or at the provider’s discretion. Scores were classified by severity: minimal (0-4), mild (5-9), moderate (10-14), moderately severe (15-19), and severe (20+) [27]. The depressive symptom cohort had baseline PHQ-9 scores of 10 or higher, which prior work validated against a trained interviewer’s assessments with 88% sensitivity and specificity [27]. Changes in symptoms between the first visit’s assessment and the last visit’s assessment were calculated and reported continuously as well as categorized as a minimal clinically important difference (MCID) defined as a reduction in symptoms of 5 or more points among those starting with a score of 10 or higher [28,29].

#### Symptoms of Anxiety

The GAD-7, a validated and reliable measure of symptoms of anxiety (Cronbach α=0.92) [30], was administered to patients upon intake at the start of treatment as well as at every third visit or at the provider’s discretion. Scores were classified by severity as previously validated: minimal (0-4), mild (5-9), moderate (10-14), and severe (15+) [30]. The anxiety symptom cohort had baseline GAD-7 scores of 10 or higher, which prior work validated against a trained interviewer’s assessments with 88% sensitivity and 82% specificity [31]. Changes in symptoms between the first visit’s assessment and the last visit’s assessment were calculated and reported continuously as well as categorized as an MCID (reduction in symptoms of 4 or more points) among those starting with a score of 10 or higher on the GAD-7 [31].

### Statistical Analyses and Reporting

Statistical analysis was performed in May 2025. Frequency counts and percentages are reported for all categorical data, and means and SDs are reported for continuous data. Bivariate (unadjusted) associations were examined via chi-square tests to determine differences between provider characteristics selected by patients and outcomes of interest, including number of visits dichotomized into 3 or more provider visits (yes or no) in the intent-to-treat populations and change in symptom of depression and anxiety scores on the PHQ-9 and the GAD-7 quantified as having an MCID (yes or no), respectively, in the complete case populations (subsets of the intent-to-treat populations with at least 2 completed PHQ-9 or GAD-7 assessments, respectively) using chi-square tests. Logistic regression models to test associations between selected provider characteristic and each outcome of interest were run adjusted for baseline demographic (age and gender) and clinical characteristics (baseline depression or anxiety symptom scores) based on theory, prior research findings, and significant variables identified in analyses that compared those in the intent-to-treat populations who were, vs those who were not, included in the complete case populations. The primary outcome of interest was having at least 3 provider visits in each intent-to-treat population (depressive symptom and anxiety symptom cohorts), and the secondary outcomes included achieving a depressive and anxiety symptom MCID in the depressive symptom complete case cohort and anxiety symptom complete case cohort, respectively. To address the potential for selection bias, sensitivity analyses were performed using the last observation carried forward method for missing clinical outcome data. Logistic regression models were expressed as odds ratios (ORs) and 95% CI of each OR. A 2-sided *P*≤.05 was considered indicative of statistical significance. All analyses were performed using R software (version 4.2; R Project for Statistical Computing).

## Results

### Sample

**Figure 1:**
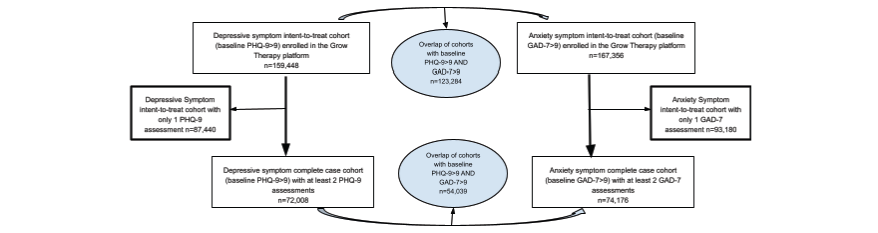
Patients with a first Grow Therapy visit in January 2022 to April 2024 with elevated baseline depressive or anxiety symptoms. GAD-7: Generalized Anxiety Disorder-7 item; PHQ-9: Patient Health Questionnaire-9 item.

The intent-to-treat depressive symptom and anxiety symptom cohorts used for engagement and sensitivity analyses consisted of 159,448 and 167,356 adult Grow Therapy patients aged 18 years or older, respectively, with elevated symptoms at baseline (PHQ-9>9 and GAD-7>9, respectively) who had a first provider visit between January 2022 and April 2024. There was a sizable overlap in the 2 intent-to-treat cohorts, with 123,284/159,448 (77.3%) of the clinically elevated depressive symptom intent-to-treat cohort also having clinically elevated anxiety symptoms, and 123,284/167,356 (73.7%) of the clinically elevated anxiety symptom intent-to-treat cohort also having clinically elevated depressive symptoms at baseline. The complete case samples used for clinical symptom analyses required at least 2 PHQ-9 or 2 GAD-7 assessments to enable the computation of a change score and included 72,008 patients in the depressive symptom cohort (72,008/159,448, 45.2% of the corresponding intent-to-treat sample) and 74,176 patients in the anxiety symptom cohort (74,176/167,356, 44.3% of the corresponding intent-to-treat sample). Again, there was a sizable overlap in the 2 intent-to-treat cohorts, with 54,039/72,008 (75%) of the clinically elevated depressive symptom complete case cohort also having clinically elevated anxiety symptoms and 54,039/74,176 (72.9%) of the clinically elevated anxiety symptom complete case cohort also having clinically elevated depressive symptoms at baseline (Figure 1).

### Baseline Patient Characteristics, Provider Selection, and Differences Between the Intent-to-Treat and Complete Case Cohorts

In the intent-to-treat cohorts, females comprised the majority of patients (about 75% in each cohort), and patients had a mean age of 33.3 (SD 11.2) years and 33.0 (SD 10.8) years in the depressive and anxiety symptom cohorts, respectively. Provider specialty was selected by approximately 35% of both cohorts, and about 5% of both cohorts selected a provider identity before the first visit (Table 1). Those in the complete case sample were overrepresented by females, had a higher mean age, and had slightly more severe depressive symptoms but slightly less severe anxiety symptoms at baseline, although the differences were not clinically significant (depressive symptom cohort completers mean baseline PHQ-9 of 15.2, SD 4.1, vs noncompleters mean baseline PHQ-9 of 15.3, SD 4.2, and anxiety symptom cohort completers mean baseline GAD-7 of 14.9, SD 3.4, vs noncompleters mean baseline GAD-7 of 15.0, SD 3.4, *P*<.001 for each comparison).

**Table 1:** Baseline characteristics and provider selections made among Grow Therapy adult patients entering in January 2022 to April 2024.

Variable						Intent-to-treat clinically elevated depressive symptom cohort (n=159,448)			Complete case clinically elevated depressive symptom cohort (n=72,008)			Intent-to-treat clinically elevated anxiety symptom cohort (n=167,356)			Complete case of clinically elevated anxiety symptom cohort (n=74,176)
**Baseline sociodemographic characteristics**															
	**Gender, n (%)**														
		Female			117,229 (73.5)			54,334 (75.5)			124,884 (74.6)			56,857 (76.7)	
		Male			41,108 (25.8)			17,167 (23.8)			41,422 (24.8)			16,855 (22.7)	
		Other			1111 (0.7)			507 (0.7)			1050 (0.6)			464 (0.6)	
	Age (years), mean (SD)				33.3 (11.2)			34.2 (11.8)			33.0 (10.8)			33.8 (11.4)	
	**Age group (years), n (%)**														
		18-24			36,191 (22.7)			15,122 (21)			37,456 (22.4)			15,520 (20.9)	
		25-34			65,783 (41.3)			28,641 (39.8)			70,888 (42.4)			30,386 (41)	
		35-44			33,570 (21.1)			15,691 (21.8)			35,691 (21.3)			16,315 (22)	
		45-54			14,365 (9)			7226 (10)			14,505 (8.7)			7087 (9.6)	
		55-64			6639 (4.2)			3624 (5)			6268 (3.7)			3355 (4.5)	
		65+			2900 (1.8)			1704 (2.4)			2548 (1.5)			1513 (2)	
**Provider characteristic selected by patient, n (%)**															
	Clinical specialty				56,094 (35.2)			25,982 (36.1)			59,573 (35.6)			27,121 (36.6)	
	Identity				8279 (5.2)			4005 (5.6)			8652 (5.2)			4069 (5.5)	
**Baseline clinical outcomes**															
	**Clinical outcome: depressive symptoms**														
		Initial depressive symptom score (PHQ-9), mean (SD)			15.3 (4.2)			15.2 (4.1)			13.6 (5.6)			13.5 (5.5)	
		**Initial depressive symptom (PHQ-9) severity category, n (%)**													
			Minimal (0-4)	0 (0)			0 (0)			7255 (4.4)			3217 (4.4)		
			Mild (5-9)	0 (0)			0 (0)			35,133 (21.2)			15,500 (21)		
			Moderate (10-14)	79,762 (50)			36,437 (50.6)			52,782 (31.9)			23,828 (32.3)		
			Moderately severe (15-19)	51,446 (32.3)			23,255 (32.3)			43,856 (26.5)			19,640 (26.7)		
			Severe (20-27)	28,240 (17.7)			12,316 (17.1)			26,647 (16.1)			11,499 (15.6)		
			Unknown	0 (0)			0 (0)			1683 (1)			492 (0.6)		
	**Clinical outcome: anxiety symptoms**														
		First anxiety symptom score (GAD-7), mean (SD)			13.6 (4.8)			13.5 (4.8)			14.9 (3.4)			14.9 (3.4)	
		**First anxiety symptom (GAD-7) severity category, n (%)**													
			Minimal (0-4)	5288 (3.3)			2420 (3.4)			0 (0)			0 (0)		
			Mild (5-9)	29,697 (18.8)			13,644 (19.1)			0 (0)			0 (0)		
			Moderate (10-14)	50,978 (32.2)			23,466 (32.8)			81,818 (48.9)			36,698 (49.5)		
			Severe (15-21)	72,308 (45.7)			32,067 (44.8)			85,538 (51.1)			37,478 (50.5)		
			Unknown	1,177 (0.7)			411 (0.5)			0 (0)			0 (0)		

The intent-to-treat samples included all adult Grow Therapy patients aged 18 years or older with a first provider visit in January 2022 through April 2024 who had clinically elevated depressive symptoms at baseline (PHQ-9 of 10-27; baseline depressive symptom cohort) or who had clinically elevated anxiety symptoms at baseline (GAD-7 of 10-21; baseline anxiety symptom cohort) as indicated.

A total of 123,284/159,448 (77.3%) of the clinically elevated depressive symptom intent-to-treat cohort also had clinically elevated anxiety symptoms and are included in both intent-to-treat cohorts.

A total of 123,284/167,356 (73.7%) of the clinically elevated anxiety symptom intent-to-treat cohort also had clinically elevated depressive symptoms at baseline and are included in both intent-to-treat cohorts.

The complete case samples included patients in each intent-to-treat sample who also had at least 2 completed PHQ-9 (baseline depressive symptom cohort) or GAD-7 assessments (baseline anxiety symptom cohort).

A total of 54,039/72,008 (75%) of the clinically elevated depressive symptom complete case cohort also had clinically elevated anxiety symptoms and are included in both complete case cohorts.

A total of 54,039/74,176 (72.9%) of the clinically elevated anxiety symptom complete case cohort also had clinically elevated depressive symptoms at baseline and are included in both complete case cohorts.

### Engagement Variables

The mean number of provider visits in the intent-to-treat depressive and anxiety symptom cohorts was 11.4 (SD 16.0) and 11.3 (SD 15.7), respectively. In the depressive symptom cohort, 69.4% had at least 3 provider visits, and 28.7% had at least 12 provider visits. In the anxiety symptom cohort, 69.3% had at least 3 provider visits, and 28.6% had at least 12 provider visits. As expected, the complete case cohorts had higher levels of engagement metrics than the intent-to-treat cohorts (Table 2).

**Table 2:** Engagement and clinical outcomes among Grow Therapy adult patients entering in January 2022 to April 2024 with at least 2 clinical outcome assessments (complete case cohorts).

Variable					Complete case clinically elevated depressive symptom cohort^a^ (n=72,008)			Complete case clinically elevated anxiety symptom cohort^b^ (n=74,176)
**Engagement, n (%)**								
	At least 3 provider visits			68,520 (95.2)			70,771 (95.4)	
	At least 12 provider visits			37,173 (51.6)			38,325 (51.7)	
**Clinical outcome: depression symptoms, mean (SD)**								
	Last depression symptom score (PHQ-9^c^)			9.1 (6.1)			8.3 (6.1)	
	**Last depression (PHQ-9) severity category, n (%)**							
		Minimal (0-4)	19,511 (27.1)			23,731 (32.8)		
		Mild (5-9)	20,875 (29)			21,192 (29.3)		
		Moderate (10-14)	17,519 (24.3)			15,133 (20.9)		
		Moderately severe (15-19)	9427 (13.1)			8153 (11.3)		
		Severe (20-27)	4676 (6.5)			4170 (5.8)		
		Unknown	N/A^d^			1797		
	Depression symptom improvement (PHQ-9), mean (SD)			6.1 (6.1)			5.2 (6.2)	
	Depression symptom MCID^e,f^, n (%)			42,392 (58.9)			32,714 (60.5)	
**Clinical outcome: anxiety symptoms**								
	Last anxiety symptom score (GAD-7^g^), mean (SD)			8.8 (5.8)^b^			9.1 (5.7)	
	**Last anxiety (GAD-7) severity category, n (%)**							
		Minimal (0-4)	19,087 (27.4)			18,496 (24.9)		
		Mild (5-9)	21,379 (30.7)			22,552 (30.4)		
		Moderate (10-14)	16,034 (23)			18,074 (24.4)		
		Severe (15-21)	13,117 (18.8)			15,054 (20.3)		
		Unknown	2391			N/A		
	Anxiety symptom improvement (GAD-7), mean (SD)			4.7 (5.9)^b^			5.8 (5.7)	
	Anxiety symptom MCID^f^, n (%)			33,705 (55.2)^b^			46,715 (63)	

^a^In the depression cohort, 71,602 had a valid GAD-7 assessment at the first (ie, baseline) visit and 69,618 had a valid GAD-7 assessment at the last (ie, second or later) visit. A total of 54,046 had a GAD-7 at baseline of 10 or greater and a valid last GAD-7 assessment to enable calculation of minimal clinically important difference.

^b^In the anxiety cohort, 73,684 had a valid PHQ-9 assessment at the first (ie, baseline) visit, and 72,379 had a valid PHQ-9 assessment at the last (ie, second or later) visit. A total of 54,040 had a PHQ-9 at baseline of 10 or greater and a valid last PHQ-9 assessment to enable calculation of the minimal clinically important difference.

^c^PHQ-9: Patient Health Questionnaire-9 item scale.

^d^N/A: not applicable.

^e^MCID: minimal clinically important difference.

^f^Minimal clinically important difference is defined as a reduction of at least 5 points on the PHQ-9 or at least 4 points on the GAD-7 between first and last Grow Therapy visit, and calculated only among those with a baseline score of 10 or greater in each cohort.

^g^GAD-7: Generalized Anxiety Disorder-7 item scale.

The intent-to-treat samples included all adult Grow Therapy patients aged 18 years or older with a first provider visit in January 2022 through April 2024 who had clinically elevated depressive symptoms at baseline (PHQ-9 of 10-27; baseline depressive symptom cohort) or who had clinically elevated anxiety symptoms at baseline (GAD-7 of 10-21; baseline anxiety symptom cohort) as indicated.

A total of 123,284/159,448 (77.3%) of the clinically elevated depressive symptom intent-to-treat cohort also had clinically elevated anxiety symptoms and are included in both intent-to-treat cohorts.

A total of 123,284/167,356 (73.7%) of the clinically elevated anxiety symptom intent-to-treat cohort also had clinically elevated depressive symptoms at baseline and are included in both intent-to-treat cohorts.

The complete case samples included patients in each intent-to-treat sample who also had at least 2 completed PHQ-9 (baseline depressive symptom cohort) or GAD-7 assessments (baseline anxiety symptom cohort).

A total of 54,039/72,008 (75%) of the clinically elevated depressive symptom complete case cohort also had clinically elevated anxiety symptoms and are included in both complete case cohorts.

A total of 54,039/74,176 (72.9%) of the clinically elevated anxiety symptom complete case cohort also had clinically elevated depressive symptoms at baseline and are included in both complete case cohorts.

### Clinical Outcomes in the Complete Case Cohorts

In the complete case depressive symptom cohort, mean PHQ-9 declined from 15.2 (SD 4.1) to 9.1 (SD 6.1) from baseline to last assessment (average change of 6.1, SD 6.1, points; Table 2). In this cohort, 58.9% (95% CI 58.5%-59.2%) of patients had a PHQ-9 depressive symptom MCID. In the complete case anxiety symptom cohort, mean GAD-7 declined from 14.9 (SD 3.4) to 9.1 (SD 5.7) from baseline to last assessment (average change of 5.8, SD 5.7, points). In this cohort, 63% (95% CI 62.6%-63.3%) of patients had a GAD-7 anxiety symptom MCID. We did not use multiple imputation (MI) because greater than 50% of eligible adults had missing outcomes data, which also was not missing at random. Prior research indicates MI is unreliable when missingness exceeds 30% and the missing-at-random assumption is violated [32,33].

### Relationship of Patient Self-Selection of a Provider Specialty to Engagement (in the Intent-to-Treat Cohorts) and Clinical Outcomes (in the Complete Case Cohorts)

Unadjusted chi-square and adjusted logistic regression models indicated no significant associations between provider specialty selection and having at least 3 provider visits in both the depressive and anxiety symptom cohorts (Table 3). Patients in the depressive symptom cohort who did select a provider specialty, however, were about 8% more likely to have a depressive symptom MCID than those who did not (59.9% vs 58.3%, OR 1.08, 95% CI 1.04-1.11). Similarly, patients in the anxiety symptom cohort who selected a provider specialty were about 4% more likely to have an anxiety symptom MCID than those who did not (63.4% vs 62.7%, OR 1.04, 95% CI 1.01-1.07), although this association was only significant at *P*<.05 in the adjusted regression analysis (*P*<.001) and not the chi-square unadjusted analysis (*P*=.07).

**Table 3:** Unadjusted and adjusted analyses predicting having at least 3 provider visits, MCID^a^ change in depressive symptoms, and MCID change in anxiety symptoms from the type of provider selection among Grow Therapy adult patients entering in January 2022 to April 2024.

			Value, n (%)	*P* value	Adjusted logistic regression β (95% CI)
**Model 1: at least 3 provider visits among patients with elevated baseline depressive symptom intent-to-treat cohort (n=159,448)**					
	**Selected provider specialty**			.67	0.99 (0.97-1.01)
		Yes	38,992/56,094 (69.5)		
		No	71,736/103,354 (69.4)		
	**Selected provider identity**			<.001	1.14 (1.08-1.20)
		Yes	5966/8279 (72.1)		
		No	104,762/151,169 (69.3)		
**Model 2: depressive symptom MCID among the elevated baseline depressive symptom complete case cohort (n=72,008)**					
	**Selected provider specialty**			<.001	1.08 (1.04-1.11)
		Yes	15,553/25,982 (59.9)		
		No	26,839/46,026 (58.3)		
	**Selected provider identity**			.57	0.98 (0.92-1.05)
		Yes	2340/4005 (58.4)		
		No	40,052/68,003 (58.9)		
**Model 3: at least 3 provider visits among Grow Therapy patients with elevated baseline anxiety symptom cohort (n=167,356)**					
	**Selected provider specialty**			.63	1.00 (0.97-1.02)
		Yes	41,334/59,573 (69.4)		
		No	74,659/107,783 (69.3)		
	**Selected provider identity**			.001	1.11 (1.06-1.17)
		Yes	6196/8652 (71.6)		
		No	109,797/158,704 (69.2)		
**Model 4: anxiety symptom MCID among elevated baseline anxiety symptom complete case cohort (n=74,176)**					
	**Selected provider specialty**			.07	1.04 (1.01-1.07)
		Yes	17,197/27,121 (63.4)		
		No	29,518/47,055 (62.7)		
	**Selected provider identity**			.07	0.95 (0.89-1.01)
		Yes	2508/4069 (61.6)		
		No	44,207/70,107 (63.1)		

^a^MCID: minimal clinically important difference.

The intent-to-treat samples used for the engagement models (models 1 and 3) included all adult Grow Therapy patients aged 18 years or older with a first provider visit in January 2022 through April 2024 who had clinically elevated depressive symptoms at baseline (PHQ-9 of 10-27; baseline depressive symptom cohort) or who had clinically elevated anxiety symptoms at baseline (GAD-7 of 10-21; baseline anxiety symptom cohort) as indicated.

A total of 123,284/159,448 (77.3%) of the clinically elevated depressive symptom intent-to-treat cohort also had clinically elevated anxiety symptoms and are included in both intent-to-treat cohorts.

A total of 123,284/167,356 (73.7%) of the clinically elevated anxiety symptom intent-to-treat cohort also had clinically elevated depressive symptoms at baseline and are included in both intent-to-treat cohorts.

The complete case samples used for the clinical symptom models (models 2, 3, 5, and 6) included patients in each intent-to-treat sample who also had at least 2 completed PHQ-9 (baseline depressive symptom cohort) or GAD-7 assessments (baseline anxiety symptom cohort).

A total of 54,039/72,008 (75%) of the clinically elevated depressive symptom complete case cohort also had clinically elevated anxiety symptoms and are included in both complete case cohorts.

A total of 54,039/74,176 (72.9%) of the clinically elevated anxiety symptom complete case cohort also had clinically elevated depressive symptoms at baseline and are included in both complete case cohorts.

Models adjusted for patient age (all models), gender (all models), baseline depression symptoms (all models), baseline anxiety symptoms (all models), and number of visits (models 2 and 4).

Unadjusted comparisons were done via chi-square tests.

### Relationship of Patient Self-Selection of a Provider Identity to Engagement (in the Intent-to-Treat cohorts) and Clinical Outcomes (in the Complete Case Cohorts)

Unadjusted chi-square and adjusted logistic regression models indicated that patients in both the depressive and anxiety symptom cohorts who selected a provider identity characteristic before the first visit were 14% and 11% more likely, respectively, to have at least 3 provider visits (72.1% vs 69.3%, OR 1.14, 95% CI 1.08-1.20, in the depressive symptom cohort and 71.6% vs 69.2%, OR 1.11, 95% CI 1.06-1.17) as compared to patients who did not select a provider identity characteristics. There were no significant unadjusted or adjusted associations, however, between the selection of a provider identity characteristic and having a depressive or anxiety symptom MCID in either cohort (Table 3).

### Sensitivity Analyses

Sensitivity analyses using the last observation carried forward for clinical outcomes in the intent-to-treat cohorts confirmed our main findings with a few exceptions. In unadjusted analyses, patients who selected both specialty and identity provider characteristics before enrollment were more likely to have a depressive symptom MCID in the depressive symptom cohort and an anxiety symptom MCID in the anxiety symptom cohort (*P*<.01 in each instance). These findings differed from the unadjusted main analyses which just found significant unadjusted associations between choosing the specialty of the provider and depressive symptom MCID in the depressive symptom cohort. The significance of the adjusted analyses, however, remained the same, with only those who selected a provider specialty, but not a provider identity, characteristic having a significantly greater likelihood of having a depressive symptom and an anxiety symptom MCID in the depressive symptom and anxiety symptom cohorts, respectively.

## Discussion

### Principal Results

In this study, a little over a third of adult patients in each cohort selected a provider specialty, and about 5% of each cohort selected a provider identity characteristic. Secondary, exploratory analyses, however, indicated that a patient’s self-selection of each provider characteristic option was associated with engagement or clinical outcomes (see Table 4 for a summary of significant findings). These findings suggest the potential value of a patient self-selecting their desired provider characteristics in order to improve their therapeutic experience and subsequent outcomes. In this study, patients who selected a provider specialty were more likely to have a change in symptoms meeting the MCID criteria in both the depressive and anxiety symptom cohorts. These findings, while preliminary and exploratory, suggest that patients who knew their presenting issues and requested targeted help from providers might have been able to experience significantly better clinical outcomes by selecting providers based on self-awareness of their treatment needs. Furthermore, patients who selected a provider identity characteristic were more likely to have at least 3 provider visits than those who did not. Selecting a provider identity characteristic was not, however, associated with clinical outcomes in either the depressive or anxiety symptom cohort. Future studies with more rigorous designs that include an appropriate control group should seek to understand the impact of extended treatment beyond PHQ-9 and GAD-7 measures, including quantifying improvements in quality of life and the role of therapeutic alliance in the observed associations.

**Table 4:** Summary of significant associations between type of provider selection and outcomes of interest (engagement and changes in clinical outcomes) in adjusted analyses confirmed by sensitivity analyses.

	Cohort and outcome				
	Elevated depressive symptom cohort			Elevated anxiety symptom cohort	
Type of provider selection	Elevated depressive symptom cohort, engagement outcomes	Elevated depressive symptom cohort, depressive symptom changes	Elevated anxiety symptom cohort, engagement outcomes		Elevated anxiety symptom cohort, anxiety symptom changes
Provider specialty	—	✓	—		✓
Provider identity	✓	—	✓		—

### Comparison With Prior Work

This is the first study of its kind to report engagement and clinical outcomes of a technology-enabled platform where patients have the choice and autonomy to select characteristics of the mental health provider treating them. Likewise, it is the first study to begin to explore the ability of patients to select provider characteristics as related to the levels of engagement and clinical outcomes found. Despite prior literature that has suggested the importance of patient choice in facilitating beneficial outcomes [18], little is known about this topic specifically related to technology-enabled platforms that provide this option.

Nonetheless, prior work examining digital mental health interventions has consistently noted low engagement rates and only modest levels of efficacy [6,7,10]. Levels of patient engagement in mental health care found in the current study, however, were quite high in comparison to even the prior recommendations from systematic reviews reported in the literature; for example, despite lower levels of actual engagement, 1 meta-analysis found that a typical internet-based CBT program recommends 5-9 visits per patient [34]. The present study’s findings are notable in this context as the proportion of patients engaging in Grow Therapy care with at least 3 provider visits (≈69%) and meeting depressive and anxiety symptom MCID criteria (≈59%-63%) is similar or higher than those reported in typical structured digital CBT programs that often have high rates of drop-out. In general, these proportions of those meeting criteria for an MCID of each outcome appeared to be similar or slightly higher than interventions previously described in the literature, especially supported digital mental health interventions delivered over the internet or via an app [23,35]. It is important to note, however, that the present analyses were conducted on a real-world sample with at least enough provider visits to capture at least 2 relevant outcome assessments, and no control group was included to enable group-level comparisons. Additional studies using more rigorous designs, such as a pragmatic RCT or using a real-world synthetic control group, that also adjust for other potential confounders of the explored associations (eg, personality characteristics, education, or other factors related to both the exposure and outcome of interest), are needed to confirm these preliminary findings.

Most previously published work has focused on naturally occurring concordance rather than patient selections of provider attributes, so our findings might not generalize to these instances. The current study examines behaviors somewhat unique to digital settings where patients can intentionally select provider characteristics unconstrained by in-person geographic location or appointment availability. This type of investigation is novel as compared to earlier concordance studies that often relied on clinic assignment, administrative matching, or geographic convenience instead of the ability to freely choose provider characteristics [11,12]. A growing body of research, however, has examined hybrid or digital care systems that have used performance-based or algorithmic matching; for example, Constantino et al [13] conducted an RCT and determined that matching patients to providers based on therapist strengths improved outcomes. The present study differs because patients could choose preferred provider characteristics, some of which they might have thought were beneficial to their care, rather than an algorithmic assignment. This distinction is important, as choice-based matching may promote early therapeutic alliance, autonomy, and expectancy effects, each of which has been linked to improved engagement and treatment success [26,36,37].

Our findings also extend recent work on digital personalization frameworks that have suggested that the inclusion of tailored content, therapist profiles, and culturally responsive design may bolster outcomes when integrated into digital mental health services [38]. While personalization in previous studies has typically involved system-driven tailoring, provider selection in this study reflects a patient-driven personalization strategy, representing an underexamined but potentially scalable approach.

Finally, few large-scale naturalistic studies have examined associations between provider-selection behaviors and clinical outcomes in digital care settings at a real-world scale. This study’s sample size exceeds most published digital mental health cohorts’ sizes and provides one of the strongest empirical demonstrations to date of how patient choice operates within commercially deployed teletherapy platforms. These findings thus contribute new evidence that scalable matching systems, when embedded within a flexible platform, may relate meaningfully to both engagement and symptom change.

### Clinical Implications

Although this study’s design limits definitive conclusions, the exploratory findings provide preliminary evidence that a web-based platform that promotes patient selection of their provider could contribute to patient engagement and favorable clinical outcomes. Specifically, selecting a provider specialty was associated with significantly achieving MCID-level improvements in depressive and anxiety symptoms. Thus, the findings suggest that encouraging patients to select a provider specialty may positively impact clinical outcomes by ensuring a more targeted fit to the expertise of the provider. In addition, selecting a provider identity characteristic was associated with having 3 or more provider visits. This pattern aligns with theoretical models proposing that autonomy, personal relevance, and therapeutic fit enhance treatment continuation [26,36,38]. The significant association between selecting a provider identity and increased patient engagement may have resulted from patients feeling more comfortable from the onset of the patient-provider relationship due to the therapeutic alliance formed between the patient and provider, promoting cultural safety and trust [11,39]. It is somewhat surprising, however, that we did not find a significant association between the selection of provider identity and changes in clinical outcomes, potentially because of the small sample size of those selecting a provider identity or perhaps due to the current study’s design itself.

Additional studies that include control groups of patients who do not select provider characteristics and that adjust for more potential confounders would lead to a greater understanding of the preliminary signals found by these exploratory analyses. These findings suggest that scalable, technology-enabled patient-provider matching systems, particularly those that allow patients to filter by provider specialty or identity, may offer a feasible way to improve engagement and outcomes at scale. As platforms such as Grow Therapy expand, this approach could be further developed to support systems–level innovation in care delivery, particularly by personalizing access in ways that are difficult to replicate in traditional models of mental health referral and triage.

Although causality cannot be inferred, these findings underscore opportunities for designing more effective digital matching interfaces. For instance, platforms may consider highlighting evidence-based specialty tags, guiding patients toward providers with relevant expertise, or using A/B testing to evaluate which provider profiles maximize engagement. Similar approaches are increasingly used in digital triage and hybrid care systems [14,40].

### Future Research Needs

In addition to the aforementioned need for more rigorous study designs to confirm these initial findings, future studies, such as pragmatic randomized or quasi-experimental studies, could also explore other potential reasons for the levels of engagement and clinical outcome success experienced by patients included in this study. For example, additional work is needed to explore potential reasons for the levels of engagement and clinical outcomes other than the selection of provider characteristics of specialty and identity. Further research is also needed to ensure that levels of outcomes are similar across different patient subgroups other than those defined by age, gender, and baseline symptomatology. These groups could be created based on social determinants of health or other vulnerable populations to ensure the equitable delivery, receipt, and experience of mental health care.

Additional work should also examine the mechanisms by which provider-selection behaviors and symptom changes are associated, perhaps via improved engagement or therapeutic alliance. Prior studies suggest that early engagement and therapeutic alliance can shape downstream clinical trajectories [28]. Research should also explore more granular subtypes of specialty and identity selection, such as having a culturally aligned provider, and whether this impacts findings. Greater attention to equity-centered matching is critical, especially as digital platforms expand access to diverse populations [23]. The current findings suggest the potential benefit of such alignment, but small sample sizes limited its evaluation. More robust investigations with larger sample sizes are needed to confirm these initial findings and to further explore more nuanced specialty and identity selections (eg, specific specialties or identity characteristics) in relation to longer-term engagement and outcomes.

### Limitations

This study has several limitations. First, our clinical outcome analyses relied on complete case data rather than using MI, which introduces potential selection bias. We deemed MI inappropriate because of substantial missing data (>50%) and nonrandom missingness after considering the evidence that MI can be invalid under these conditions [32,33]. Thus, clinical outcome findings may be generalizable only to patients who completed at least 2 PHQ-9 or GAD-7 assessments. Sensitivity analyses using last observation carried forward, however, yielded similar adjusted findings, increasing confidence in the robustness of observed associations. As reasons for missingness were not captured, we could not determine whether those lost to follow-up were dissatisfied with their treatment, realized early symptom improvement so did not “need” treatment anymore (“happy abandonment” [41]), or if some other, unobserved factors were in play.

Second, due to limited sample sizes for hundreds of possible provider specialty and identity combinations, this study examined only binary selections rather than specific match types (eg, trauma specialty; Black/African American identity). Future research with larger subgroup samples may explore more granular associations. Additional limitations include reliance on observational data without a control group, lack of adjustment for unmeasured confounders such as socioeconomic status or provider experience, and potential overlap between depressive and anxiety symptom cohorts. We also did not examine how engagement and outcomes relate to each other mechanistically; future studies should test whether engagement mediates clinical change. Additional studies to determine the amount and types of engagement needed to achieve beneficial outcomes, including whether engagement mediates the associations with clinical outcomes, will provide a better understanding of the findings of this study. Finally, because analyses did not adjust for multiple comparisons, findings should be interpreted as exploratory.

Third, a majority of the sample was in both elevated baseline symptoms of depression and anxiety cohorts, given the high prevalence of comorbid baseline symptoms, rendering the engagement analyses somewhat duplicative. The reported regression analyses, however, did report associations adjusted for both baseline depressive and anxiety symptoms. We did not, however, have access to other potentially confounding variables such as socioeconomic status, education, or provider experience to further adjust the models. These factors should be included in future research. Additionally, we did not adjust the analyses for multiple comparisons, given the exploratory nature of this study. Most reported significant associations, however, were at the *P*<.001 level of significance that would have remained significant even after adjustment.

Fourth, although this study examined the patient-driven selection of providers, the Grow Therapy platform’s interface, available provider pool, and algorithmic ordering of provider profiles may influence what options users see and select. These platform-level design factors were not examined but may partially explain patterns of selection and should be considered in future research [42].

Fifth, this study did not evaluate therapist characteristics beyond those made available to patients for selection (eg, provider experience, caseload, and therapeutic fidelity). Prior work suggests that therapist effects can meaningfully influence outcomes and unmeasured provider-level variability may confound associations [43].

Finally, measures of patient preference strength (eg, how strongly a patient valued a specific attribute) were not collected. Prior meta-analytic work suggests that preference strength, rather than preference presence alone, may predict outcomes [17].

More recent quality improvement initiatives targeting measurement-informed care, which promotes the completion and use of routine outcome monitoring as part of the treatment relationship between a provider and a patient [44], have improved clinical symptom measure completion rates at Grow Therapy since the sample’s inclusion of earlier data periods (2022-2024). Future outcome analyses that include these more robust samples will thus serve to enhance the generalizability of findings, as would the use of a study design that includes an appropriate control group.

### Conclusions

In this large-scale real-world analysis, patients who selected provider characteristics before initiating care demonstrated meaningful differences in both engagement and clinical outcomes. While exploratory, these findings contribute new evidence that patient autonomy in selecting provider attributes, a feature uniquely enabled by digital mental health platforms, may play an important role in shaping therapeutic trajectories. This is likely the first real-world study of the associations between different types of provider selections and engagement and clinical symptom changes in a digital mental health platform for patients with elevated symptoms of depression or anxiety. Further research using more rigorous controlled designs is needed to confirm these preliminary findings and better understand how types of and more nuanced provider selection variables, such as specialty (eg, depression, anxiety, trauma, etc) and identity (Black/African American, Muslim, etc), are associated with engagement or clinical symptom outcomes. As the field continues to evolve toward more personalized models of digital care [19], understanding how patients navigate matching tools and how these behaviors relate to outcomes will be critical for developing scalable, equitable, and effective mental health services.

## Data Availability

The datasets generated or analyzed during this study are available from the corresponding author on reasonable request.

## Abbreviations

CBT: cognitive behavioral therapy
GAD-7: Generalized Anxiety Disorder-7 item
HIPAA: Health Insurance Portability and Accountability Act
MCID: minimal clinically important difference
MI: multiple imputation
OR: odds ratio
PHQ-9: Patient Health Questionnaire-9 item
RCT: randomized controlled trial
STROBE: Strengthening the Reporting of Observational Studies in Epidemiology
